# The direct-vision stenting method: an innovative stent placement technique using a novel thin scope for malignant gastric outlet obstruction

**DOI:** 10.1055/a-2621-3035

**Published:** 2025-07-10

**Authors:** Teruyuki Numata, Tomohiro Shimada, Taku Yamagata, Yoshihide Kanno, Takeshi Shimizu, Kento Hosokawa, Kei Ito

**Affiliations:** 1 Department of Gastroenterology, Sendai City Medical Center, Sendai, Japan

A 54-year-old male with pancreas head cancer had previously undergone biliary metal stent placement. He later developed malignant gastric outlet obstruction (mGOO) around the superior duodenal angle ( Fig. 1 ), necessitating endoscopic duodenal stenting. However, careful stent placement was required to avoid interference with the existing biliary stent, which could lead to biliary stent dysfunction or unexpected duodenal injury.

**Fig. 1:**
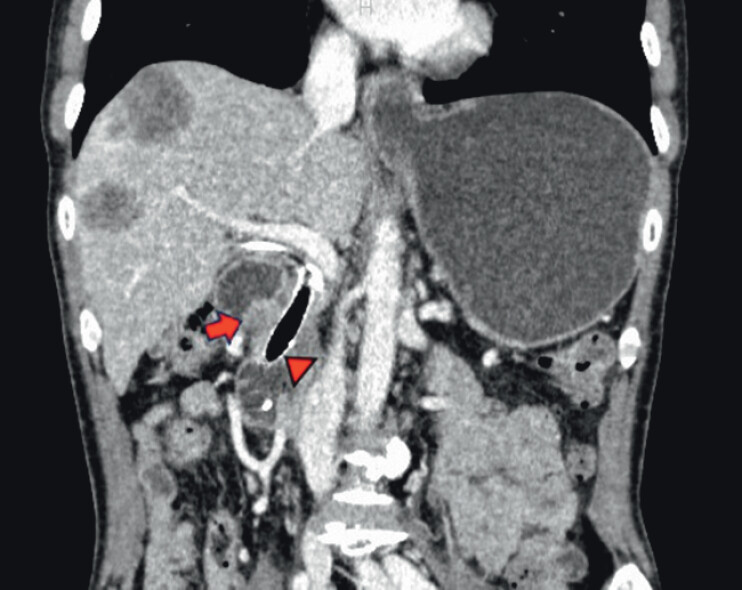
Contrast-enhanced computed tomography shows the malignant gastric outlet obstruction around the superior duodenal angle (arrow) caused by pancreatic head cancer. Arrow head, a biliary stent previously placed.

To achieve optimal stent positioning, we used a newly developed thin therapeutic scope, the EG-840TP scope (Fujifilm, Tokyo, Japan) 1 ( Fig. 2 ). This scope features a 7.9-mm outer diameter, a 3.2-mm accessory channel, a waterjet function, and sufficient deflection capabilities, including a 160-degree downward angulation. The scope was easily advanced into the anal side of the obstruction without resistance. Under endoscopic visualization, the 22-mm metal stent (Hanarostent; Boston Scientific Japan K.K., Tokyo, Japan) was strategically deployed in stages: first, partially expanded within the anal-side lumen to confirm safe positioning relative to the biliary stent, then gradually expanded to cover the stenotic segment with a direct-vision stenting (DVS) method 2 ( Video 1 , Fig. 3 ). This approach minimized the risk of stent interference, and the obstructive symptoms were relieved without adverse events.

**Fig. 2:**
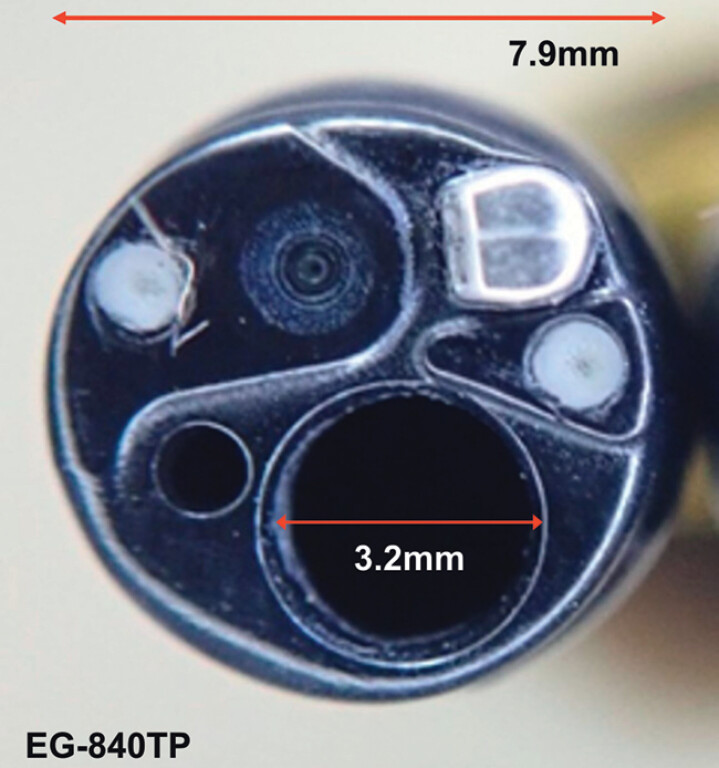
Front-view image of the thin therapeutic endoscope EG-840TP. This scope features an outer diameter of 7.9 and a 3.2 mm accessory channel.

The direct-vision stenting method using the EG-840TP scope for malignant gastric outlet obstruction.Video 1

**Fig. 3:**
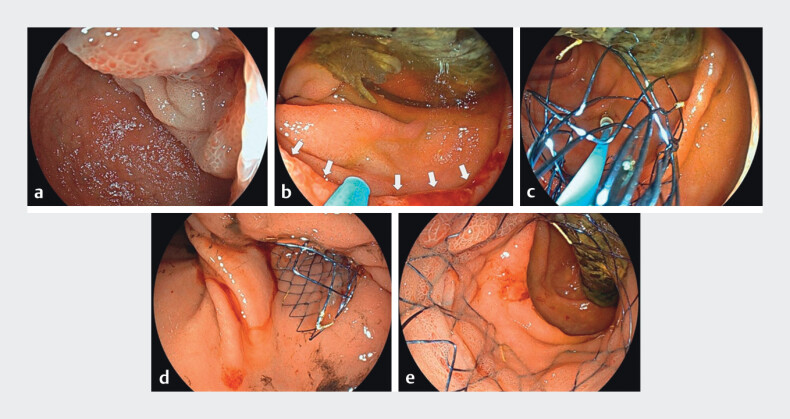
Stent placement using the EG-840TP scope. **a** A tight stricture was observed at the superior duodenal angle. **b** After the scope advanced through the stricture, the biliary metal stent was observed. Arrows, anal edge of the malignant stricture. **c** The stent was partially expanded within the anal-side lumen under the endoscopic view, to confirm safe positioning. **d, e** The intestinal stent was placed at the precise position without affecting the biliary stent.

Endoscopic stent placement with the conventional through-the-scope technique is effective 3 , but the anal lumen cannot be visualized, making it challenging to assess how the intestinal wall or existing stents are affected. Moreover, precise identification of the anal edge of the stricture is difficult, requiring longer stents. The DVS method addresses these issues, enabling placement of shorter stents that precisely fit the stricture and preserve the option for future biliary reinterventions. While feasibility remains uncertain, further research is required.

Endoscopy_UCTN_Code_TTT_1AO_2AZ

## Notes

Endoscopy E-Videos https://eref.thieme.de/e-videos
*E-Videos* is an open access online section of the journal *Endoscopy* , reporting on interesting cases and new techniques in gastroenterological endoscopy. All papers include a high-quality video and are published with a Creative Commons CC-BY license. Endoscopy E-Videos qualify for HINARI discounts and waivers and eligibility is automatically checked during the submission process. We grant 100% waivers to articles whose corresponding authors are based in Group A countries and 50% waivers to those who are based in Group B countries as classified by Research4Life (see: https://www.research4life.org/access/eligibility/ ). This section has its own submission website at https://mc.manuscriptcentral.com/e-videos .
